# Heel hidradenocarcinoma regarded as a corn for 25 years with subsequent multiorgan metastasis: a case report and literature review

**DOI:** 10.3389/fonc.2026.1914842

**Published:** 2026-08-14

**Authors:** Lianbo Yang, Ziang Zheng, Haoran Chen, Xinyao Cui, Jiabin Lin, Yifan Wang, Haidong Liang, Jing Yi

**Affiliations:** Department of Hand and Foot Surgery and Bone and Soft Tissue Repair and Reconstruction, Second Affiliated Hospital of Dalian Medical University, Dalian, China

**Keywords:** case report, diagnostic delay, foot neoplasms, heel, hidradenocarcinoma, metastasis, molecular profiling

## Abstract

**Background:**

Hidradenocarcinoma is a rare malignant cutaneous adnexal tumor with nonspecific clinical features. Heel involvement is uncommon and may be mistaken for a benign hyperkeratotic or pressure-related lesion.

**Case presentation:**

A 44-year-old woman was referred in 2020 with a non-healing left heel lesion that, according to retrospective history, had been regarded as a corn for approximately 25 years. After ulceration and failure to heal following laser treatment, extended excision was performed. Histopathological examination supported hidradenocarcinoma, and all surgical margins were negative. Approximately 2.5 years later, a left inguinal mass developed. Pathological examination showed a poorly differentiated carcinoma consistent with hidradenocarcinoma, with lymphovascular tumor emboli and findings suspicious for lymph-node metastasis. Estrogen receptor expression was negative, progesterone receptor showed weak positivity in fewer than 1% of tumor cells, and HER2 amplification was not detected by fluorescence *in situ* hybridization. Nab-paclitaxel plus cisplatin achieved stable disease after four cycles. Subsequent gemcitabine plus capecitabine was accompanied by progression, grade 3 anemia, and grade 2 thrombocytopenia. Skeletal and intracranial metastases were identified in early 2024, after which the patient was lost to follow-up.

**Conclusion:**

Persistent, enlarging, ulcerated, recurrent, or treatment-resistant acral lesions warrant timely pathological assessment. Complete excision remains central to localized disease, whereas regional staging, radiotherapy, systemic treatment, and molecularly guided therapy should be individualized because supporting evidence remains limited.

## Introduction

Hidradenocarcinoma is a rare malignant cutaneous adnexal neoplasm showing sweat-gland differentiation. Historical terminology includes malignant hidradenoma, malignant nodular hidradenoma, clear-cell hidradenocarcinoma, and malignant acrospiroma. In a systematic review of 165 published cases, the median age at presentation was 61 years, and lower-extremity tumors accounted for approximately 15% of reported cases (1).

Clinical recognition is difficult because hidradenocarcinoma lacks a characteristic appearance. It may present as a slowly enlarging nodule, plaque, cyst-like mass, ulcer, or recurrent wound and may therefore be mistaken for a benign cutaneous process. Definitive diagnosis relies primarily on histopathological examination, with immunohistochemistry used to support epithelial or adnexal differentiation and exclude important mimics (2, 3).

The clinical behavior of hidradenocarcinoma is variable. The published-case systematic review found that 36.2% of patients presented with or subsequently developed metastatic disease (1), whereas a population-based SEER analysis of 289 patients reported a 10-year cancer-specific survival rate of 90.5% (4). These differences reflect biological heterogeneity as well as case-report publication bias, inconsistent terminology, and incomplete follow-up.

Complete surgical excision with histologically negative margins remains the principal treatment for localized disease. The optimal excision margin and the roles of sentinel lymph-node biopsy, lymphadenectomy, radiotherapy, chemotherapy, endocrine therapy, targeted therapy, and comprehensive molecular profiling remain uncertain (1, 5).

Heel and plantar presentations are uncommon but not unprecedented Previous reports have documented heel hidradenocarcinoma with ipsilateral inguinal nodal metastases, a medial ankle tumor with a prolonged clinical history, and low-grade plantar hidradenocarcinoma with lymph-node metastasis (6–8). The present case is notable for an approximately 25-year patient-reported history, subsequent ipsilateral inguinal disease, and later skeletal and intracranial metastases. Because serial images, biopsies, and molecular specimens were unavailable during the prolonged prediagnostic interval, the case is interpreted as prolonged diagnostic uncertainty rather than proof of tumor dormancy or a distinct biphasic biological transition.

## Case report

A 44-year-old woman was referred to our hospital in 2020 for a non-healing lesion of the left heel. According to retrospective history, the lesion had been clinically regarded as a corn approximately 25 years earlier and had not undergone pathological examination. No clinical photograph or dermoscopic image from the pre-ulceration period was available.

In May 2020, the lesion ulcerated, measuring approximately 1.0 × 1.0 cm. It persisted after laser treatment at another institution and subsequently enlarged. An excisional biopsy performed on October 28, 2020, was initially reported as a malignant mixed tumor of the skin. Persistent non-healing of the surgical wound prompted referral to our hospital.

**Figure 1:**
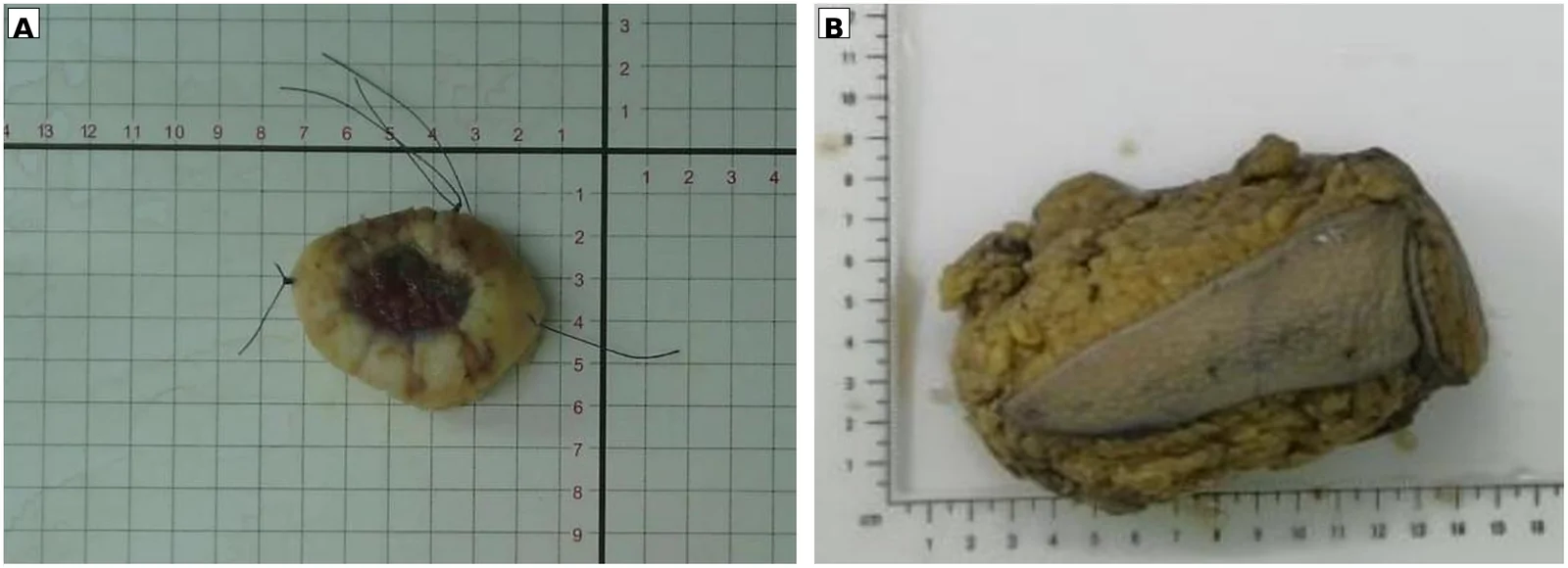
Gross specimens. **(A)** Resected primary tumor from the left heel; the black line outlines the tumor margin. **(B)** Gross specimen from the subsequently resected left inguinal mass.

**Figure 2:**
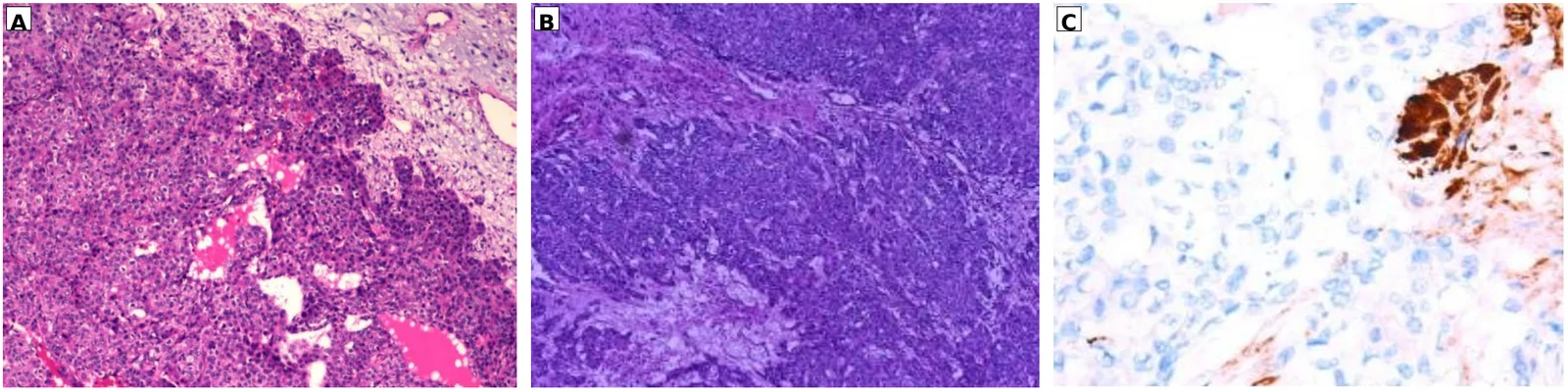
Representative histopathological and immunohistochemical findings. **(A)** Hematoxylin and eosin (H&E) staining of the primary heel tumor showing solid nests of atypical epithelial cells with cytological atypia. **(B)** H&E staining of the left inguinal tumor showing a nodular proliferation of poorly differentiated carcinoma with surrounding lymphoid and fibrous stroma. In conjunction with the previous clinical and pathological history, these findings were considered consistent with hidradenocarcinoma. **(C)** Progesterone receptor immunohistochemistry of the left inguinal tumor showing focal nuclear staining in fewer than 1% of tumor cells. The brown-stained cell cluster in the upper-right portion of the panel represents PR-positive tumor cells.

On November 18, 2020, an extended local excision was performed. The specimen measured 6 × 5 × 2 cm and contained an ulcerated area measuring 3 × 2.4 cm (**Figure 1A**). Histopathological examination supported a diagnosis of hidradenocarcinoma. The primary heel tumor was composed predominantly of solid nests of atypical epithelial cells infiltrating the deep dermis and subcutaneous tissue (**Figure 2A**). The tumor cells showed abundant cytoplasm, nuclear pleomorphism, prominent nucleoli, and mitotic activity. Immunohistochemically, the tumor cells were positive for AE1/AE3, CAM5.2, and CK7 and negative for GCDFP-15, S-100, HMB45, and CD31. The Ki-67 proliferation index was approximately 40%. All surgical margins were negative.

The postoperative defect was reconstructed using a full-thickness skin graft harvested from the ipsilateral inguinal region. Postoperative care included wound and graft monitoring, pressure off-loading, pain assessment, mobility support, and patient education.

**Figure 3:**
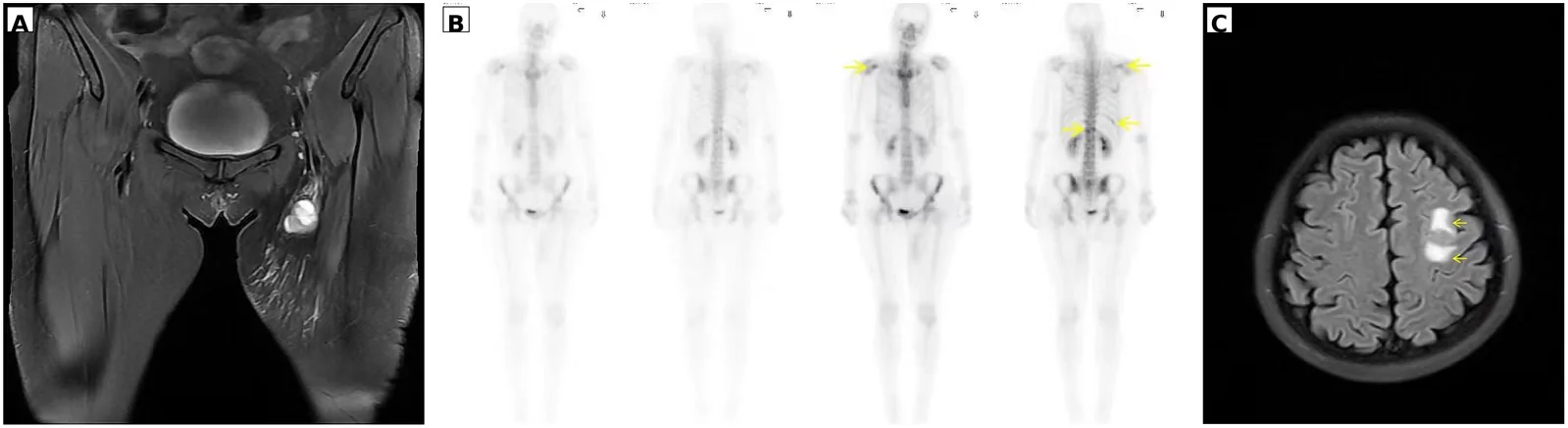
Imaging findings during disease progression. **(A)** Contrast-enhanced pelvic magnetic resonance imaging showing a heterogeneously enhancing cystic-solid mass in the left inguinal region. **(B)** Whole-body bone scintigraphy showing new radiotracer uptake in the right scapula, right posterior 10th rib, and T12 vertebra. **(C)** Contrast-enhanced brain magnetic resonance imaging showing the displayed left frontal metastatic lesion; a left cerebellar metastatic lesion was also reported radiologically but is not displayed in this panel.

Approximately 2.5 years after the primary operation, the patient presented in April 2023 with a mass in the left proximal medial thigh/inguinal region. Magnetic resonance imaging showed a lobulated cystic-solid lesion measuring approximately 34 × 31 × 51 mm, with restricted diffusion and heterogeneous enhancement, raising suspicion for a malignant tumor or metastatic lymph node (**Figure 3A**).

The patient underwent resection of the left inguinal mass with regional lymph-node dissection. The resected specimen measured 18.5 × 9 × 5 cm and contained a 4 × 3.5 × 3 cm gray-white mass within the subcutaneous tissue. The mass was focally cystic and contained blood clot-like material. Histopathological examination showed a poorly differentiated carcinoma that, in conjunction with the previous clinical and pathological history, was considered consistent with hidradenocarcinoma. The tumor exhibited a nodular growth pattern with abundant surrounding lymphoid and fibrous tissue (**Figure 2B**), raising suspicion for lymph-node metastasis. Lymphovascular tumor emboli were present, whereas perineural invasion was not identified. The skin, circumferential, and deep resection margins were negative for tumor. The available pathology report did not specify the total number of dissected lymph nodes, the number of involved nodes, or extranodal-extension status.

From July to November 2023, the patient received nab-paclitaxel plus cisplatin. Radiological assessment after four cycles showed stable disease. To explore potentially targetable treatment options, the left inguinal tumor was evaluated for ER, PR, and HER2 status. These breast-associated therapeutic biomarkers were assessed because sweat-gland carcinomas may occasionally express hormone receptors or demonstrate HER2 amplification; the testing was not intended to diagnose breast carcinoma.ER expression was negative, whereas PR immunohistochemistry showed focal nuclear staining in fewer than 1% of tumor cells (**Figure 2C**). HER2 amplification was not detected by FISH; the mean HER2 copy number was 2.0 signals per cell, the mean CEP17 copy number was 1.6 signals per cell, and the HER2/CEP17 ratio was 1.25. These findings did not support endocrine or HER2-directed treatment.

Comprehensive next-generation sequencing was not performed during the patient’s clinical course. Because this is a retrospective case report, the accessible medical records did not document a specific reason for not pursuing molecular profiling. Following disease progression, gemcitabine plus capecitabine was initiated and was complicated by grade 3 anemia and grade 2 thrombocytopenia.

In January 2024, whole-body bone scintigraphy revealed new lesions involving the right scapula, right posterior 10th rib, and T12 vertebra, which were considered consistent with skeletal metastases (**Figure 3B**). Brain magnetic resonance imaging in February 2024 demonstrated metastatic lesions in the left frontal lobe and left cerebellar hemisphere (**Figure 3C**). Maintenance capecitabine was prescribed, and palliative cranial radiotherapy was recommended. Supportive care during systemic therapy and subsequent metastatic progression focused on treatment-related toxicity, pain and neurological symptoms, functional safety, and coordination of oncology and radiotherapy follow-up. The patient was subsequently lost to follow-up after February 2024; it could therefore not be determined whether cranial radiotherapy was administered, and subsequent treatment response, survival, and end-of-life outcomes were unavailable. The major clinical events are summarized in **Table 1**.

**Table 1:** Clinical timeline.

Date	Clinical event
Approximately 1995	Left heel lesion was reportedly regarded as a corn; no biopsy or contemporaneous clinical image was available.
May 2020	Ulceration developed; the lesion persisted after laser treatment and enlarged.
October 28, 2020	Excisional biopsy at another institution was initially reported as a malignant mixed tumor of the skin.
November 18, 2020	Extended local excision confirmed hidradenocarcinoma with negative margins; the defect was reconstructed with a full-thickness skin graft.
April-May 2023	Left proximal medial thigh/inguinal disease was detected and resected with regional lymph-node dissection.
July-November 2023	Nab-paclitaxel plus cisplatin was administered; stable disease was documented after four cycles.
Late 2023	Gemcitabine plus capecitabine was administered after progression and was complicated by hematological toxicity.
January-February 2024	Bone and brain metastases were identified; cranial radiotherapy was recommended.
After February 2024	The patient was lost to follow-up.

## Discussion

This case is primarily a diagnostic warning. A persistent acral lesion that enlarges, ulcerates, bleeds, drains, recurs, or fails to heal should raise suspicion for an underlying malignancy and prompt timely pathological evaluation, even after a prolonged period of apparent clinical stability. In this patient, the 25-year duration and earlier label of corn were reconstructed retrospectively. Because no pre-ulceration photograph, dermoscopic image, biopsy, or serial pathological specimen was available, the original appearance and biological status of the lesion cannot be independently verified.

The heel location likely contributed to diagnostic difficulty because hyperkeratotic and pressure-related lesions are common at weight-bearing sites, whereas malignant adnexal tumors are rare. Previously reported hidradenocarcinomas or malignant hidradenomas involving the heel, sole, foot, toes, and ankle are summarized in **Table 2**. These cases demonstrate markedly heterogeneous clinical courses, ranging from prolonged localized disease to regional lymph-node metastasis, distant dissemination, and disease-related death. The present patient had a longer patient-reported period of benign clinical labeling than most reported cases and later developed sequential inguinal, skeletal, and intracranial disease. However, the available data do not confirm tumor dormancy, immune equilibrium, or a discrete transition from an indolent to an aggressive biological phase.

**Table 2:** Published hidradenocarcinoma or malignant hidradenoma cases involving the heel, sole, foot, toes, or ankle.

Report	Age/Sex	Site	Duration	Clinical features	Treatment	Outcome
Delgado et al., 2003 (9)	41/F	Left foot	Not reported	Clinically node-negative; inguinal sentinel node 0/1	Primary surgery and sentinel lymph-node biopsy	Alive without disease during reported short follow-up
Bogner et al., 2003 (10)	78/F	Right foot	Not reported	Tumor 9 × 12 mm; 1/5 sentinel nodes positive; subsequent regional nodes 0/13	Primary excision, sentinel lymph-node biopsy, and regional lymphadenectomy	No evidence of disease at 25 months
Kazakov et al., 2009 (3)	34/F	Sole	Not reported	Local recurrence after initial excision	Excision	Recurrence at 4 years; no evidence of disease at 11 years
Kazakov et al., 2009 (3)	84/M	Sole, foot, and lower leg	20 years	Poorly demarcated sole tumor with multiple nodular or bruise-like lesions; possible cerebral metastasis	Treatment declined	Died of disease 5 months after diagnosis
Kazakov et al., 2009 (3)	59/F	Left heel	8 years	Large heel tumor; detailed nodal staging not reported in the series table	Below-knee amputation	No evidence of disease at 7 years
Nazarian et al., 2009 (2)	62/F	Right fifth toe	Not reported	Malignant hidradenoma; tumor present at the specimen margin	Excision	Follow-up unavailable
Nazarian et al., 2009 (2)	62/F	Left foot	Not reported	Malignant hidradenoma measuring 1.4 cm	Excision	No evidence of disease at 2 years 2 months
Labbardi et al., 2017 (6)	64/M	Right heel	2 years	Ulcerated 5-cm tumor with ipsilateral inguinal lymph-node metastases and capsular rupture	Wide excision, inguinal lymphadenectomy, and radiotherapy	No recurrence at 34 months
Kane et al., 2018 (7)	85/M	Left medial ankle	15 years	Fungating hypervascular lesion; no reported nodal or distant metastasis	Surgical excision and reconstruction	No recurrence at 18 months
Roman et al., 2023 (8)	33/F	Left plantar foot	5 years	Low-grade 3 × 3-cm tumor with a small popliteal lymph-node metastasis	Excisional biopsy and sentinel lymph-node biopsy	Lost to follow-up
Present case	44/F	Left heel	Approximately 25 years by retrospective history	Ipsilateral inguinal disease followed by skeletal and intracranial metastases	Extended excision, grafting, inguinal surgery, and two chemotherapy regimens	Lost to follow-up after brain metastases

Historical terminology includes malignant hidradenoma, malignant nodular hidradenoma, clear-cell hidradenocarcinoma, and malignant acrospiroma. Some pathological or molecular publications may include overlapping patients; this table is intended for qualitative clinical comparison rather than patient-level meta-analysis.

Histopathological assessment remains central to diagnosis. Features supporting malignancy include poor circumscription, infiltrative growth, extension into deeper tissues, nuclear pleomorphism, increased mitotic activity, tumor necrosis, lymphovascular invasion, and perineural invasion. These findings are not uniformly present and should be interpreted as an integrated architectural and cytological pattern rather than as isolated criteria (2, 3). In the present primary tumor, infiltrative solid nested architecture and cytological atypia (**Figure 2A**), together with prominent nucleoli, mitotic activity, an epithelial immunophenotype, and a Ki-67 proliferation index of approximately 40%, supported the diagnosis of hidradenocarcinoma.

Published estimates of metastatic risk vary. The systematic review by Kent et al. reported metastatic disease in 36.2% of published cases, with regional lymph nodes being the most frequent metastatic site (1). The SEER analysis by Gao et al. reported a 10-year cancer-specific survival rate of 90.5% (4). Hidradenocarcinoma should therefore be described as clinically heterogeneous rather than uniformly aggressive or rapidly fatal.

The ipsilateral inguinal disease in this patient is anatomically consistent with expected lymphatic drainage from a heel malignancy. A previous report of heel hidradenocarcinoma with ipsilateral inguinal metastases supports this interpretation (6). Although the skin graft used in the initial reconstruction was harvested from the ipsilateral inguinal region, temporal and anatomical association alone does not establish iatrogenic implantation. Natural lymphatic dissemination adequately explains the observed pattern, and this case does not justify a general recommendation for routine contralateral or distant graft-donor-site selection.

Complete surgical excision with histologically negative margins remains the most consistently recommended treatment for localized hidradenocarcinoma (1, 5). Mohs micrographic surgery may provide complete peripheral and deep margin assessment in selected anatomically suitable tumors, but available retrospective comparisons are vulnerable to differences in site, size, stage, and follow-up. The role of sentinel lymph-node biopsy also remains unsettled. Delgado et al. and Bogner et al. showed that lymphatic mapping and sentinel-node biopsy are feasible in sweat-gland carcinomas, including hidradenocarcinoma, but these small heterogeneous studies do not demonstrate a survival benefit or mandate routine use in every patient (9, 10).

Adjuvant radiotherapy has been used for positive or close margins, recurrent disease, perineural invasion, poor differentiation, and nodal involvement. Harari et al. reported postoperative irradiation of malignant sweat-gland neoplasms using approximately 70 Gy to the primary bed and approximately 50 Gy to regional lymphatic basins; the small historical series suggested possible locoregional control but could not establish an overall-survival benefit (11).

No standardized systemic regimen has been established for unresectable or metastatic hidradenocarcinoma. Published treatments include platinum compounds, taxanes, anthracyclines, fluoropyrimidines, gemcitabine, and targeted agents, with inconsistent and generally anecdotal responses (1, 5). Capecitabine has shown activity in an individual metastatic case (12), and sunitinib produced temporary disease control in reported patients with metastatic skin adnexal carcinomas, including hidradenocarcinoma, after prior chemotherapy (13). In the present patient, nab-paclitaxel plus cisplatin achieved stable disease after four cycles, whereas gemcitabine plus capecitabine was accompanied by progression and clinically relevant hematological toxicity. These observations illustrate therapeutic uncertainty but cannot establish comparative efficacy.

ER, PR, and HER2 were assessed to explore potentially targetable treatment options rather than to establish the diagnosis. Sweat-gland carcinomas may occasionally express breast-associated therapeutic biomarkers, but the findings are heterogeneous and do not justify automatic application of a breast-cancer treatment algorithm. HER2 amplification has been reported in metastatic hidradenocarcinoma (14), whereas other series found amplification to be uncommon (3). In this patient, ER was negative, PR staining involved fewer than 1% of tumor cells, and HER2 amplification was absent, providing no support for endocrine or HER2-directed therapy.

Molecular findings in hidradenocarcinoma remain incompletely characterized. Studies of apocrine-eccrine and metastasizing adnexal carcinomas have identified heterogeneous alterations, including TP53 and PIK3CA mutations in limited subsets (15, 16). These data support consideration of comprehensive molecular profiling in selected patients with advanced or treatment-refractory disease when adequate tissue, access, and clinical circumstances permit. At present, however, no recurrent actionable alteration or biomarker-directed standard treatment has been established, so next-generation sequencing should be presented as an individualized exploratory option rather than a mandatory component of routine management.

This case also underscores the need for stage-appropriate supportive care after heel reconstruction and during advanced disease. Because nursing interventions and patient-reported outcomes were not prospectively collected, these observations are descriptive rather than evidence of intervention effectiveness.

This report has several limitations. It describes a single patient; the long duration and earlier impression of a corn were based on retrospective patient history; serial pathological and molecular specimens were unavailable during the prolonged prediagnostic interval; a formal mitotic count per 10 high-power fields was not available; complete nodal counts and extranodal-extension status were not documented in the accessible report; comprehensive next-generation sequencing was not performed; and loss to follow-up prevented assessment of cranial radiotherapy delivery, subsequent treatment response, overall survival, and end-of-life outcomes.

## Conclusion

Hidradenocarcinoma should be considered in the differential diagnosis of a persistent, enlarging, ulcerated, recurrent, or non-healing heel lesion, even when it has long been regarded as benign. This case documents ipsilateral inguinal disease followed by skeletal and intracranial metastases after margin-negative excision and illustrates the limited evidence supporting systemic treatment in advanced disease.

The available data do not prove tumor dormancy, a biphasic biological transition, or iatrogenic tumor implantation at the graft donor site. The most defensible clinical implications are timely biopsy of changing acral lesions, detailed pathological documentation, individualized regional staging, cautious use of biomarker and molecular testing, coordinated multidisciplinary care, and structured follow-up.

## Data Availability

The original contributions presented in the study are included in the article/supplementary material. Further inquiries can be directed to the corresponding authors.
